# Principles for Developing a Large-Scale Point-of-Care Ultrasound Education Program: Insights from a Tertiary University Medical Center in Israel

**DOI:** 10.5334/pme.1613

**Published:** 2025-05-22

**Authors:** Roy Rafael Dayan, Ofri Karni, Itamar Ben Shitrit, Rachel Gaufberg, Karny Ilan, Lior Fuchs

**Affiliations:** 1 Faculty of Health Sciences, Ben-Gurion University of the Negev, Israel; 2 Clinical Research Center, Soroka University Medical Center, Beer-Sheva, Israel; 3 General Surgery Department, Sheba Medical Center, Israel; 4 Intensive Care Unit, Soroka University Medical Center,Beer-Sheva, Israel

## Abstract

**Background & Need for Innovation:**

Point-of-care ultrasound (POCUS) has transformed bedside diagnostics, yet its operator-dependent nature and lack of structured training remain significant barriers. To address these challenges, Ben Gurion University (BGU) developed a longitudinal six-year POCUS curriculum, emphasizing early integration, competency-based training, and scalable educational strategies to enhance medical education and patient care.

**Goal of Innovation:**

To implement a structured and scalable POCUS curriculum that progressively builds technical proficiency, clinical judgment, and diagnostic accuracy, ensuring medical students effectively integrate POCUS into clinical practice.

**Steps Taken for Development and Implementation:**

The curriculum incorporates hands-on training, self-directed learning, a structured spiral approach, and peer-led instruction. Early exposure in physics and anatomy courses establishes a foundation, progressing to bedside applications in clinical years. Advanced technologies, including AI-driven feedback and telemedicine, enhance skill retention and address faculty shortages by providing scalable solutions for ongoing assessment and feedback.

**Evaluation of Innovation:**

Since its implementation in 2014, the program has trained hundreds of students, with longitudinal proficiency data from over 700 students. Internal studies have demonstrated that self-directed learning modules match or exceed in-person instruction for ultrasound skill acquisition, AI-driven feedback enhances image acquisition, and early clinical integration of POCUS positively influences patient care. Preliminary findings suggest that telemedicine-based instructor feedback improves cardiac ultrasound proficiency over time, and AI-assisted probe manipulation and self-learning with ultrasound simulators may further optimize training without requiring in-person instruction.

**Critical Reflection:**

A structured longitudinal approach ensures progressive skill acquisition while addressing faculty shortages and training limitations. Cost-effective strategies, such as peer-led instruction, AI feedback, and telemedicine, support skill development and sustainability. Emphasizing clinical integration ensures students learn to use POCUS as a targeted diagnostic adjunct rather than a broad screening tool, reinforcing its role as an essential skill in modern medical education.

## Background & Need for Innovation

In the past decade, point-of-care ultrasound (POCUS) has gained immense popularity and utility, emerging as an invaluable diagnostic tool at the bedside for clinicians worldwide [1]. Its portability, ease of use, and ability to provide immediate and actionable insights that affect clinical decision-making have led to its introduction to daily patient care [2]. Given its extensive range of benefits, the application of POCUS has enhanced patient evaluation, diagnosis, and management in a variety of healthcare contexts, including those with limited resources, rural regions, and isolated areas [3,4,5,6].

With the increasing availability of affordable ultrasound (US) systems, POCUS is recognized by some as the ‘fifth element’ of bedside clinical examination, complementing inspection, palpation, percussion, and auscultation [7]. Consequently, several organizations, including the Accreditation Council for Graduate Medical Education and the International Consensus Conference on Ultrasound Education for Undergraduate Medical Students, have established foundational guidelines for ultrasound education [1,8]. As a result, POCUS has become an integral part of medical training, spanning medical school, residency, and fellowship programs [9,11,31,32].

Ultrasound serves as a valuable learning tool from the early stages of medical education, reinforcing anatomy and physiology through real-time visualization while bridging theoretical knowledge with practical imaging [7,12]. Early exposure fosters technical skill development and pattern recognition, with studies showing that US proficiency can be acquired quickly and improves with practice [9,13]. It also strengthens bedside assessment by complementing rather than replacing physical examinations, addressing the decline in traditional examination skills [7,14,15,16]. Additionally, since US improves procedural accuracy and safety, integrating it into procedural training allows students to develop safer techniques and a deeper understanding of its role in reducing complications [1,17].

Despite the growing integration of POCUS into medical training, its widespread adoption faces significant barriers. A survey across multiple institutions highlighted key obstacles, including limited access to structured training, inadequate supervision, time constraints during clinical rotations, and a lack of quality assurance frameworks [10]. Additionally, institutional barriers such as faculty shortages and insufficient funding further restrict POCUS education [10]. Apart from these structural constraints, POCUS is inherently operator-dependent, requiring structured training to ensure high-quality imaging and accurate clinical interpretation. Without proper education, its misuse—particularly as a general screening tool—can lead to false findings, misdiagnoses, and potential patient harm [18,19,20,21]. These challenges underscore the need for a structured, longitudinal curriculum that systematically builds competency from early training stages.

## Goal of Innovation

**Figure 1:**
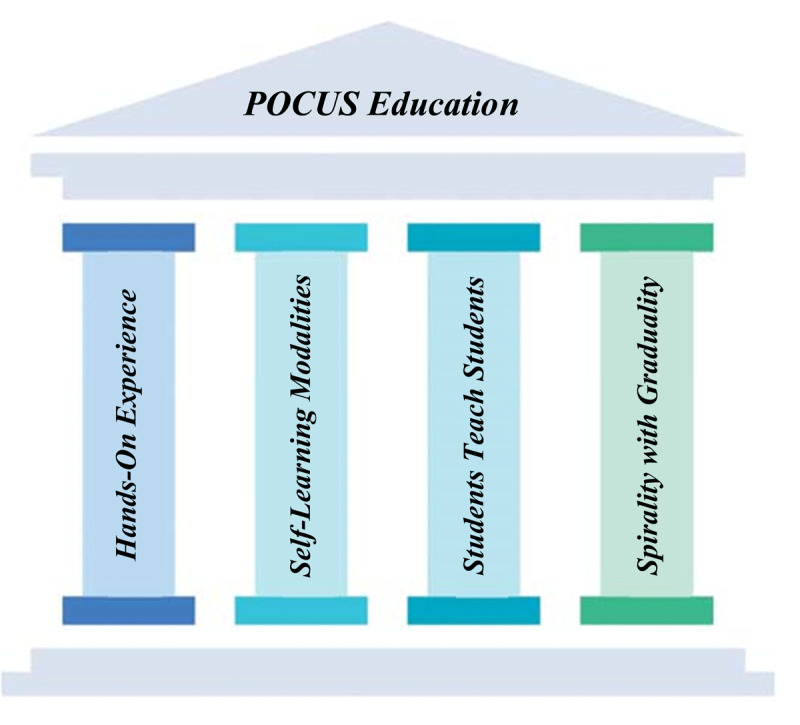
Our Four Pillars of Our POCUS Education Program.

To address the barriers in POCUS education, a structured, longitudinal six-year curriculum has been developed, emphasizing early integration and progressive skill acquisition. This framework is built upon four foundational pillars: hands-on experience, self-learning modalities, peer-teaching and spirality with graduality (Figure 1). Designed as a scalable solution, this model optimizes learning resources, advocating early integration for competency development, and enhances accessibility while working within institutional constraints. By leveraging these principles, medical students are equipped with the technical skills and clinical judgment necessary to integrate POCUS effectively into patient care, bridging the existing knowledge gap and fostering a sustainable approach to ultrasound training.

## Steps Taken for Development and Implementation of Innovation

### Early Integration is Key

**Figure 2:**
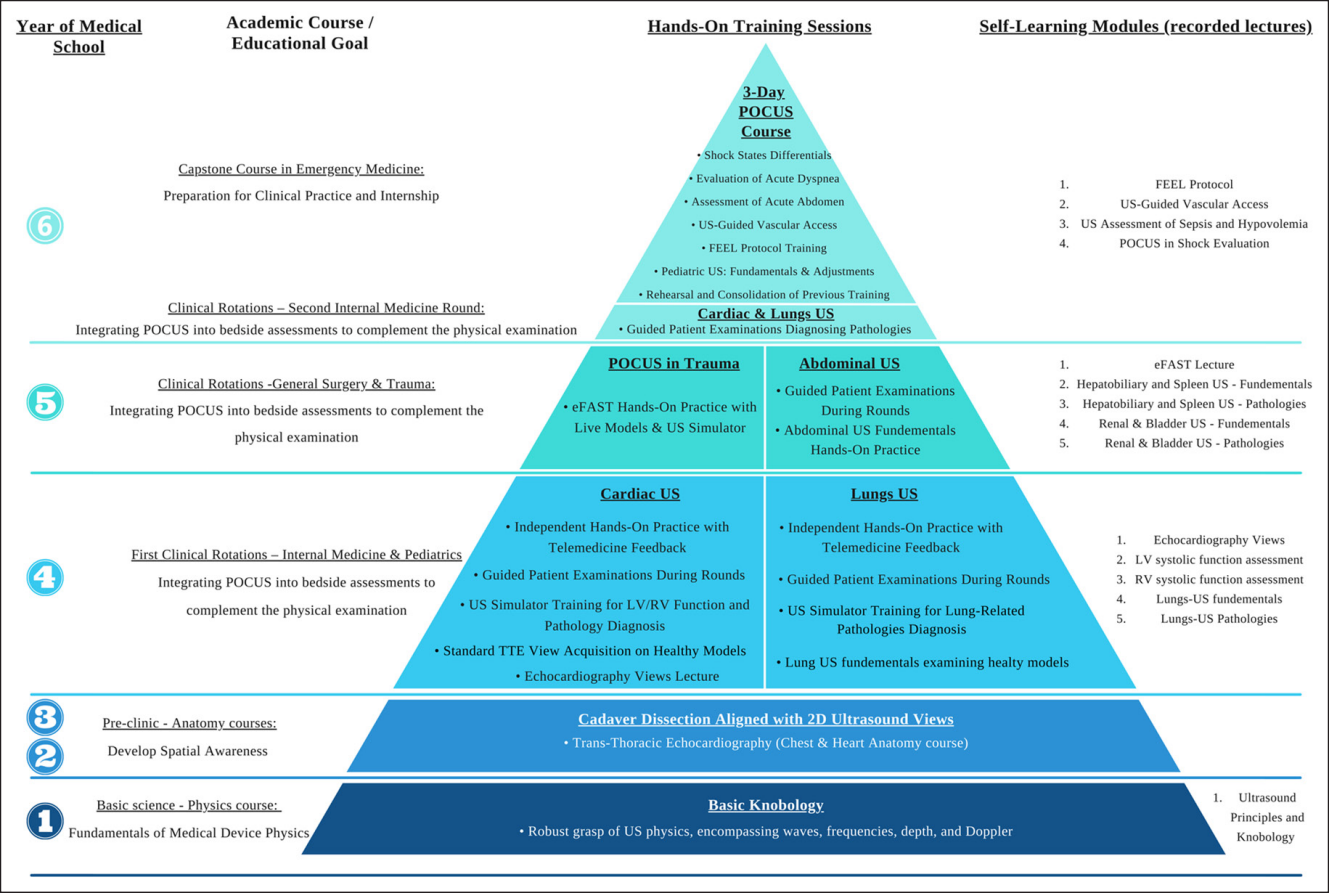
Spirality with Graduality teaching steps via the syllabus pyramid. *The chart is structured from left to right, outlining the six-year medical school program. Each academic course is underlined, with its corresponding educational goal, relating to POCUS, indicated beneath. Within the pyramid chart, each • represents a single academic hour dedicated to hands-on training (except for telemedicine, which is detailed separately below). On the far right of the chart, a list details pre-recorded lectures that students are required to self-learn in preparation for hands-on training sessions on the respective subject. For example, during the fourth year, as part of their first clinical rotations in internal medicine and pediatrics, students attend a single in-person lecture on echocardiography views. This is followed by six hours of hands-on training with guided instruction, covering cardiac and lungs POCUS, conducted in small groups with a 6:1 student-to-instructor ratio. To further reinforce skill development, students are also required to submit POCUS examinations they perform during clinical rounds for instructor feedback via telemedicine. Abbreviations: LV (Left Ventricle), RV (Right Ventricle), eFAST (Extended Focused Assessment with Sonography in Trauma), FEEL (Focused Echocardiography in Emergency Life Support), POCUS (Point-of-Care ultrasound), TTE (Trans Thoracic Echocardiography), US (ultrasound).

A structured, longitudinal integration of ultrasound from the first year of medical school ensures progressive skill acquisition and reinforces learning at each stage of education. By introducing US in preclinical courses such as physics and anatomy, students develop spatial awareness and imaging proficiency early, establishing a foundation for clinical application [7,12]. This early exposure facilitates a smooth transition into bedside assessments, where US complements physical examination by enhancing diagnostic accuracy [1,7,14,15,16]. As training advances, US is further integrated into procedural instruction, reinforcing its role in improving safety and precision during interventions [1,17]. This structured approach ensures that US learning evolves alongside clinical education, preparing students to incorporate POCUS effectively into patient care (Figure 2). A study from our program further supports this model, demonstrating that students who engaged in early US training exhibited greater confidence, technical proficiency, and diagnostic accuracy when utilizing POCUS in clinical settings [22].

### 1. Hands-On Experience

Hands-on training has been recognized as a preferred approach for skill-based learning, particularly in US education [23,24,25,26]. Given its effectiveness in developing technical proficiency [24], a longitudinal six-year POCUS curriculum should prioritize hands-on experience to ensure competency. For example, in the fourth year, students are trained to integrate bedside echocardiography into the comprehensive physical examination. This method combines an in-person didactic lecture on echocardiographic views with three structured hands-on training sessions. These sessions include echocardiographic view acquisition, a dedicated training session on assessing left and right ventricular systolic function, and a final session focused on patient examinations to identify pathological findings during clinical rounds (Figure 2). The curriculum emphasizes real-time scanning over passive learning, ensuring skill development in both simulated and clinical settings while addressing the challenge of insufficient training opportunities [10]. To mitigate supervision limitations, group sizes are restricted to a 1:6 instructor-student ratio, enhancing feedback and individualized instruction.

To reinforce hands-on learning and encourage continued skill application, telemedicine technology may be integrated into clinical training to provide remote feedback on student-performed POCUS examinations. Within the proposed curriculum, students submit echocardiography scans accompanied by a brief patient description, allowing instructors to assess image quality, probe handling, and clinical relevance. This approach fosters independent practice while maintaining structured guidance, particularly in resource-limited settings. To evaluate the effectiveness of this telemedicine-based feedback system, a study was conducted during the internal medicine rotation late in the fourth year. As part of the trial, students were required to submit three cardiac US scans from patients they examined, preferably including pathological findings, and received individualized feedback from their instructors. Performance was assessed using a cardiac ultrasound proficiency exam, with validity evidence supporting its use for evaluating medical student skills in this context [9,27]. The exam was administered before clinical rotations following two initial hands-on training sessions and reassessed four months later at the conclusion of the internal medicine round. Preliminary results indicate that students who engaged in remote feedback sessions demonstrated significantly better skill retention and higher performance scores compared to those who did not participate. These findings underscore the potential of remote supervision as a scalable strategy for maintaining POCUS proficiency, particularly in settings where regular hands-on training is not feasible.

### 2. Self-Learning Modalities

A key challenge in POCUS education is the lack of institutional funding and structured training, requiring efficient resource allocation [10]. Self-learning modules may allow educators to prioritize hands-on training by shifting theoretical instruction to independent learning. Approaches range from recorded lectures to external platforms (Figures 2, S1). Integrating self-learning with hands-on training may enhance theoretical understanding and skill acquisition. As illustrated in the previously presented example, students are instructed to review a recorded lecture on LV systolic function before the hands-on training session, allowing them to revisit the material as needed to reinforce learning before or after practical training (Figure 2).

Our studies have demonstrated the effectiveness of self-directed learning in POCUS education. One study found that students supplementing a hands-on cardiac POCUS course with e-learning achieved significantly better image acquisition, particularly in the parasternal long-axis, apical four-chamber, and inferior vena cava views, compared to those relying solely on the course [28]. Another study comparing self-directed lung US training to bedside instruction showed higher test scores and superior scanning techniques in the self-learning group, suggesting noninferiority, if not superiority, of this approach [29]. Additionally, a cohort study on cardiac US self-training found that students who combined e-learning with self-practice performed comparably to those trained in a structured, faculty-led course, reinforcing the feasibility of self-directed learning [27].

Beyond self-learning, AI-driven feedback provides an additional resource-efficient method for skill development without nessecitating an instructor. A study evaluating an AI-based real-time quality indicator tool found that students using AI feedback achieved higher performance scores and improved image quality across multiple echocardiographic views, including those where the AI tool was inactive [30]. In another trial, students equipped with a real-time AI-driven navigation tool and a quality bar indicator obtained better apical views than students who used the same POCUS device without AI features [36]. By integrating self-learning and AI-assisted training, POCUS education becomes more scalable, accessible, and resource-efficient, addressing training limitations while ensuring competency development.

### 3. Students Teach Students

As noted, universal training implementation is hindered by time constraints, faculty shortages, and limited funding [1,10,28,29]. We have adopted the known *“students teach students”* approach to address these challenges [33,34,35]. In a randomized control trial, 3rd-year medical students taught by near-peer instructors showed equal effectiveness and reassurance in POCUS education compared to faculty instructors [34]. These supports utilize near-peers as a resource-efficient strategy for high-quality POCUS instruction. In addition, a study by our group demonstrated that in an 8-hour cardiac US program, students taught by their peer teaching assistants (TAs) outperformed those taught by expert trainers in demonstrating anatomical landmarks, suggesting that classmates can effectively teach POCUS imaging acquisition [33]. As part of this initiative, we have established a program enabling 4th-year students to become POCUS instructors. Selected students, after specialized training by experts, transition into TAs. This innovative approach addresses the scarcity of expert trainers, utilizing qualified classmates as TAs to facilitate widespread POCUS education in medical schools.

### 4. Spirality with Graduality

The spiral and gradual learning approach ensures that POCUS education is seamlessly integrated across all years of medical training, allowing students to progressively build on their knowledge and refine their technical skills. This method reinforces foundational concepts while introducing increasingly complex applications, enabling students to develop proficiency in POCUS and the ability to diagnose specific pathologies (Figure 2).

The curriculum begins in the first year with an US physics course, where students learn the fundamental principles of US mechanics through didactic instruction, followed by a hands-on session to practice probe handling, depth and frequency adjustments, and image interpretation. This foundation prepares them for the second and third years, where they apply their basic knobology skills to visualize echocardiographic views using cadaver hearts and 3D cardiac models, bridging the gap between anatomical theory and practical imaging.

By the fourth year, students revisit these principles with a more advanced focus, acquiring echocardiographic views on live models before transitioning to bedside POCUS on patients during clinical rotations. Having already developed a strong foundation in cardiac US, students extend their knobology skills to lung US, progressively expanding their clinical applications. In the fifth year, POCUS training is integrated into the general surgery rotation, where students are trained to perform eFAST examination, reinforce cardiac and lung US techniques (e.g., pneumothorax assessment), and receive additional training in hepatobiliary and renal ultrasound through a recorded lecture followed by hands-on practice.

In the final year, POCUS serves as a capstone within the Emergency Medicine course, emphasizing its role in managing acutely ill patients. Training focuses on the differential diagnosis of shock mechanisms, requiring students to synthesize knowledge from cardiac function assessment, lung ultrasound, and FAST examination. Additionally, students apply the Focused Echocardiography in Emergency Life Support (FEEL) protocol during cardiac arrest scenarios, reinforcing the use of POCUS in time-sensitive decision-making. In cases of dyspnea and acute abdomen, students revisit abdominal and lung US techniques, ensuring proficiency in integrating multiple modalities for accurate diagnosis. Throughout all training sessions, emphasis is placed on correlating POCUS findings with the appropriate clinical context rather than employing it as a broad screening tool, thereby enhancing both diagnostic accuracy and patient safety.

Each stage of training builds upon prior knowledge, reinforcing concepts while progressively expanding their application, ensuring students develop both technical competency and clinical reasoning. This structured, longitudinal approach provides continuous rehearsal and skill refinement, ultimately preparing students for independent POCUS use in their future practice (Figure 2, see full description in Appendix 1).

## Evaluation of Innovation

Since its implementation in 2014, the structured six-year Ben Gurion University-Soroka POCUS curriculum has trained hundreds of students and provided longitudinal proficiency data from over 700 students. To assess its effectiveness, we conducted multiple studies using a cardiac US proficiency exam, with validity evidence supporting its use in assessing student performance within our curriculum [9,27], as well as US pathology questionnaires to evaluate competency in cardiac and lung ultrasound [29].

Our team has demonstrated that self-directed learning modules can match or exceed in-person instruction for ultrasound skill acquisition [27,28,29]. AI-driven feedback has been shown to enhance apical four-chamber view acquisition [30,36], and early integration of POCUS in clinical rounds allowed fourth-year students to positively impact patient diagnoses and management [22].

Preliminary findings suggest that telemedicine-based instructor feedback improves cardiac ultrasound proficiency after extended periods without guided training, supporting long-term skill retention. Additionally, self-directed hands-on training with POCUS simulators appears to be a viable independent learning strategy.

## Critical Reflection on The Process

Implementing a large-scale POCUS curriculum in a resource-limited environment requires balancing cost-effectiveness, faculty availability, and high-quality training. Given budget constraints, we have prioritized scalable solutions by integrating a dedicated simulation center with five POCUS devices and three mannequin simulators for pathology demonstration, as well as a web-based platform with recorded lectures (Figure S1). This approach shifts theoretical instruction to self-directed learning, allowing hands-on sessions to focus on skill acquisition.

With an average class size of 120 students, maintaining adequate supervision is a challenge. Training sessions are capped at a 1:6 instructor-student ratio, ensuring individualized feedback. To further address faculty shortages, a structured instructor training program begins in the fourth year, preparing senior students to teach fundamental knobology and image acquisition. This students-teach-students model expands instructional capacity, promotes peer learning, and reinforces the instructors’ own proficiency.

While POCUS has impressive diagnostic accuracy in the hands of skilled operators, inexperienced practitioners may misinterpret findings, potentially harming patients [19,20]. Key pitfalls justify the need for early educational programs. First, POCUS is operator-dependent, requiring proper training to ensure high-quality imaging and accurate interpretation within the clinical context. Without foundational knowledge—such as understanding machine settings, probe handling, modes, and image orientation—image quality and interpretation suffer. Second, false-positive and false-negative diagnoses may occur, particularly when POCUS is used as a general screening tool without proper clinical context [18,21]. It is essential to educate practitioners on posing relevant clinical questions before conducting examinations. Lastly, over-reliance on POCUS can lead to errors; it must complement, not replace, a comprehensive clinical evaluation and sound judgment.

A key pedagogical challenge is ensuring students use POCUS appropriately—not as a broad screening tool, but as a targeted diagnostic adjunct. To address this, we emphasize early clinical integration and ensure that advanced training—such as ultrasound in shock evaluation, dyspnea, and acute abdomen in the sixth year—is guided by experienced physicians. This structure allows students to refine their clinical reasoning while benefiting from expert feedback in complex diagnostic scenarios.

## Data Accessibility Statement

The datasets used and/or analysed during the current study are available from the corresponding author on reasonable request.

## Additional File

The additional file for this article can be found as follows:

10.5334/pme.1613.s1 Supplementary File. Supplementary Figure S1 and Appendix 1.

## Abbreviations

Point-of-Care Ultrasound (POCUS), Ben Gurion University (BGU), Artificial Intelligence (AI), Soroka University Medical Center (SUMC), Ultrasound (US), eFAST (Extended Focused Assessment with Sonography in Trauma), FEEL (Focused Echocardiography in Emergency Life Support), TTE (Trans Thoracic Echocardiography), Teaching Assistants (TAs).
