# Supplementary material for: Principles for Developing a Large-Scale Point-of-Care Ultrasound Education Program: Insights from a Tertiary University Medical Center in Israel
Source: Perspect Med Educ. 2025 May 22;14(1):319–27. doi: 10.5334/pme.1613 (PMC12101120; doi:10.5334/pme.1613)
Supplement: Supplementary File. — Supplementary Figure S1 and Appendix 1. [file pme-14-1-1613-s1.pdf]

Supplementary

Figure S1 - *web-based self-learning examples* [25]

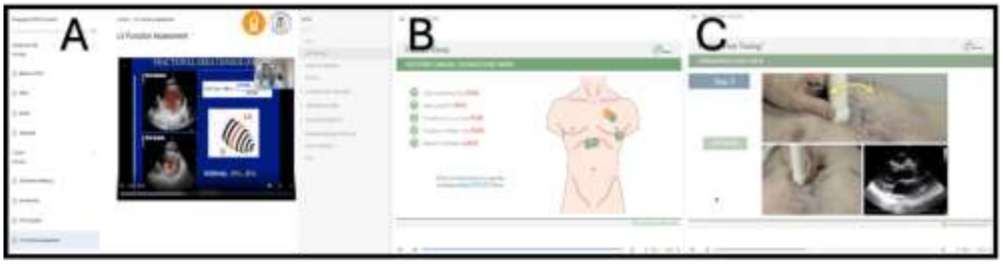

(A) Our Emergency POCUS course video lectures page (B), (C) Represent examples from the online course of eMedical Academy. Figures were taken with permission from the eMedical Academy formal web-based course (https://www.emedicalacademy.com)

Appendices

Appendix 1: Outline of POCUS Program curriculum

Timeline	Content	Required Achievements	Educational Methods
1st-year	Basic Physics course for medical students	Robust grasp of US physics, encompassing waves, frequencies, depth, and Doppler.	• A formal presentation on the fundamental physical principles associated with sound waves.<br>• Self-learning via a recorded lecture about US principles.<br>• US workshop on healthy models, demonstrating practical application of the learned physical principles during live imaging.
2nd year	General Anatomy Courses	Identification of common transthoracic cardiac views, key abdominal organs, and musculoskeletal US features.	• A didactic presentation on anatomical structures conducted in dissection rooms using cadavers for demonstration.<br>• Self-learning via a recorded lecture about echocardiography views.<br>• A didactic session on echocardiography, featuring demonstrations on either a cadaver heart or 3D heart models, in conjunction with a TTE examination conducted on a healthy

3rd year			subject.
4th-year	Pre-Clerkship Course	Acquire standard TTE views:<br>• Parasternal Long Axis (PLAX)<br>• Parasternal Short Axis (PSAX)<br>• Apical 4, 5, 2, 3 Chambers views (A4C, A5C, A2C, A3C)<br>• Subcostal and Inferior Vena Cava visualization (SC, IVC)	• A didactic session on TTE, presenting cardiac US views alongside projecting live demonstrations of the examination conducted on a healthy subject.<br>• Self-learning via recorded lectures about US principles and TTE views.<br>• Two hands-on training sessions in small groups to acquire standard TTE views on healthy models.
	First Clinical Rotations:<br>• Internal Medicine<br>• Pediatric	• Understanding that POCUS exams must be made with the appropriate clinical correlation.<br>• Performing cardiac US exams on hospitalized patients, emphasizing the accompanying challenges and the common cardiac pathologies seen in the internal wards.<br>• Perform essential lung US scans in both internal and pediatric wards, identifying B-lines, pleural effusion, consolidations, and lung sliding.	• Bedside teaching of POCUS integration as part of a comprehensive physical examination of patients.<br>• Self-learning via recorded lectures about lung US examination technique and pathologies identification.<br>• Two hands-on training sessions in small groups utilize the advanced 3D Systems Simbionix US Mentor for pathology training (Simbionix, Beit Golan, Israel) [37].<br>• Bedside hands-on in the internal ward for real-time cardiac and lung pathologies identification.<br>• Submit cardiac and lung POCUS images of patients to teaching assistants for review.
5th-year	General Surgery rotation	• Perform abdominal US examinations, identifying liver, gallbladder, bile ducts, portal vein, spleen, kidneys, bladder, abdominal aorta, and uterus.<br>• Acquiring proficiency in the eFAST examination.	• Self-learning via recorded lectures about abdominal US examination technique, pathologies, and eFAST examination.<br>• Hands-on training sessions in small groups for performing abdominal US exams and eFAST techniques on the healthy model. Additionally,

			utilize the Simbionix US Mentor for eFAST pathology training.<br>• Bedside hands-on in the general surgery ward for real-time identification of abdominal US pathologies
6th-year	Capstone Course in Emergency Medicine: Acute Care and Critical Care Training	• Employ POCUS for differential diagnosis of shock states (hypovolemic, cardiogenic, septic, obstructive including tamponade, high-risk pulmonary embolism, and tension pneumothorax).<br>• Use lung ultrasound for dyspnea assessment.<br>• Use abdominal ultrasound for abdominal pain assessment.<br>• Practice the US-guided IV access technique.<br>• Perform pediatric cardiac and lung US examinations at a basic level.<br>• Introduction to US use in OBGYN.<br>• ß support (ACLS) protocols.<br>• Apply POCUS in various clinical cases from the ICU.	• The 3-day POCUS course comprises dedicated cardiac, lungs, and abdominal POCUS hands-on sessions using healthy models and simulators, providing personalized feedback.<br>• Integrate POCUS in emergency code management scenarios for training in eFAST examinations and utilizing POCUS in FEEL protocol.<br>• Utilize vascular access models for technique demonstration and further training in US-guided vascular access on peer students.
	Second Internal Medicine round	Practice the acquired POCUS skills in assessing a patient's cardiac function and differential diagnosis of dyspnea and acute abdomen.	• Self-learning via recorded lectures about assessment of cardiac function and US examination in dyspnea cases.<br>• Bedside hands-on in the internal ward for integration of cardiac, lung, and abdominal POCUS skills

*Year of 6-year medicine school program
